# Historicizing the liberal antiracism of Cultural Evolution

**DOI:** 10.1007/s40656-024-00647-1

**Published:** 2024-12-05

**Authors:** Cameron Brinitzer

**Affiliations:** Max Planck Institute for the History of Science, Department II, Berlin, Germany

**Keywords:** Culture, Evolution, Race, Antiracism, Liberalism, Quantification

## Abstract

The Cultural Evolution Society was established in 2015 to “catalyze a theoretical synthesis” in the scientific study of human culture. As a field of research, cultural evolution took shape in the 1970s and 1980s around the aim of incorporating culture into biology’s modern evolutionary synthesis. Cultural evolution grew around the turn of the twenty-first century at the interface of population genetics and cognitive psychology. This article locates the origins of research on cultural evolution in projects of postwar scientific antiracism and U.S.-based debates about race and intelligence in the 1960s. Charting the development of prominent approaches to studying cultural evolution, I show how population geneticists and cognitive psychologists worked to redefine culture in statistical, populational, and geographic terms to politically neutralize the study of human difference. I situate the forms of genetic and cognitive culturalism that emerged as a result in a longer history of twentieth-century scientific antiracism.

## Introduction

Cultural evolution is a field of research that emerged in the late twentieth century around the aim of using the theoretical and methodological apparatus of evolutionary genetics to study human culture. The field was given its first contours by population geneticists in the 1970s, and over the course of the 1980s and 1990s, its boundaries were defined by its relations to sociobiology, evolutionary psychology, and the cognitive sciences. The field of cultural evolution expanded in the early twenty-first century, entrenching and institutionalizing a political epistemology of human difference that had been consolidated in the liberal postwar life sciences. While the field is marked by theoretical divisions and methodological disagreements, cultural evolutionists are totally unified in their ambition “to rethink the human sciences from a modern evolutionary perspective” (Brewer et al., 2017, p. 1).

Modern is key. Culture had already been an object of evolutionary thinking for a century when the field of cultural evolution began taking shape. The historical specificity of cultural evolution as it emerged in the late twentieth century lies in its constitutive aspiration to account for culture within the framework of biology’s modern evolutionary synthesis. Cultural evolution developed on the premise that extending the synthesis to account for culture was an urgent epistemic task with a full understanding of human evolution at stake. In the 1970s, several groups of population geneticists and evolutionary biologists began arguing that, just as it had only been possible to mathematically formalize Charles Darwin’s theory of natural selection after the rediscovery of Gregor Mendel’s mechanism of genetic transmission, an extension of Neo-Darwinian theory to the evolution of culture would likewise require the identification and elaboration of cultural *transmission mechanisms* (Smocovitis, 1992). As long as evolutionary scientists left culture’s distinctive transmission mechanisms unexamined, they argued, evolutionary theory would have, at best, only half the story.

The Cultural Evolution Society was established in 2015 with 600 founding members across the globe, though highly concentrated in North America and Europe (Brewer, 2016a, p. 4).^1^ The overarching aim of the new society was to “catalyze a theoretical synthesis in the study of culture” (Brewer et al., 2017, p. 1). The new society held its inaugural election in 2016. For each position on its Executive Committee, two candidates were selected through a nomination process, who then faced each other in a runoff election. The candidates for the position of the society’s first president were the American evolutionary biologist Peter Richerson and the French cognitive scientist Dan Sperber. Richerson won over 70% of the votes cast and was elected the society’s first president; Sperber was named president-elect in accordance with a special condition of the inaugural election, intended “to ensure continuity of leadership in this fledgling stage for the society” (Brewer, 2016b, p. 6).

Soon after the society’s inaugural election, its founders issued a survey to all members aimed at identifying urgent problems and research priorities for the field. After subjecting the responses to close-text semantic analysis, thematic coding and clustering, the organizers announced eight “grand challenges” for twenty-first century cultural evolution. They labeled the largest node in their cluster analysis “knowledge synthesis,” which they described as encompassing challenges relating to the theoretical synthesis of the work of different schools of cultural evolution. By 2016, the two most prominent research programs in the field were the “California School,” led by Peter Richerson and his longtime collaborator Robert Boyd, and the “Paris School,” led by Dan Sperber (Sterelny, 2017).^2^ In their discussion of the challenges facing the field, the Cultural Evolution Society’s founders raised a set of fundamental questions for their science, which reflected longstanding disagreements between the California and Paris schools: “What are the cognitive and behavioural processes underlying cultural transmission? How can we most usefully conceptualize the units of cultural transmission? What does it mean when we say that a culture evolves?” (Brewer et al., 2017, p. 3).

The theoretical disagreements that pit the Paris and California schools of cultural evolution against one another grew out of their distinct disciplinary and methodological commitments. The California school comprises evolutionary biologists who produce theories through mathematical formalizations of culture and quantitative models of cultural and genetic co-evolution, whereas the Paris school is made up of cognitive scientists who mobilize the methods of experimental psychology in laboratory settings to investigate the cognitive mechanisms of cultural transmission. Although the two school’s common project of creating a quantitative and mechanistic science of cultural evolution has bound their programs together since the turn of the twenty-first century, their enduring debates concern fundamental theoretical and methodological questions about how to define and investigate their common epistemic object.

In this article, I chart how by 2015 the field of cultural evolution came to be defined by this scientific rivalry.^3^ Although this context does not appear in cultural evolutionists’ own accounts of their field’s history, I locate the emergence of research on cultural evolution in U.S.-based debates about race and intelligence in the 1960s. While cultural evolutionists frequently tout their field’s nonracial and even antiracist orientation, I argue that it was postwar scientific antiracism’s distinct ambivalences and ambiguities which raised the question of culture’s evolution for geneticists in the first place.

## Toward a history of culturalisms across the human and life sciences

Studying culture from an evolutionary perspective is an aim as old as anthropology itself. The first European science of culture to go by the name sought to explain the origin and evolution of civilization in deterministic, naturalistic, and nomothetic terms (Tylor, 1865, 1920 [1871]). Its author, Edward B. Tylor, conceptualized culture as a universal process of rationalizing human thought and action, which progressed through three stages: from savagery to barbarism to civilization (Tylor, 1920 [1871]; Stocking, 1965, 1968, 1987). In Tylor’s definition, culture was singular and hierarchical, a matter of degree; societies had more or less of it depending on the stage in which they were located. In spite of the scientific upheavals initiated by the publication of Charles Darwin’s *On the Origin of Species* (1859), Tylor applied pre-Darwinian taxonomic methods to the study of culture, comparing archaeological and ethnographic data in order to arrange human groups on this scale of civilization which culminated in the “educated world of Europe and America” (Tylor, 1920 [1871], p. 26).^4^

Tylor articulated his science of culture as a liberal intervention in mid-nineteenth century debates about human origins. Against polygenists, who argued that different human races had evolved from distinct origins, Tylor wrote that it was “both possible and desirable to eliminate considerations of hereditary varieties or races of man, and to treat mankind as homogenous in nature, though placed in different grades of civilization” (1920 [1871], p. 7). For Tylor, the important differences between human groups were not to be found in nature but in culture. The task for a science of culture was to compare humans in different stages of civilization in order to establish a ranked “scale of races from savages to ourselves” (Tylor, 1920 [1871], p. 27). At the same time, against degenerationists who argued that “lower races” had degenerated from an original, higher, and providential state, Tylor wrote that the “main tendency of human society during its long term of existence has been to pass from a savage to a civilized state,” and that non-European races simply represented a more primitive condition of the human species (Tylor, 1920 [1871], p. 32). Culture designated the process of this progressive improvement as well as its cumulative sum at a given time and place.

A conventional historical narrative has located classic anthropologies like Tylor’s within the nineteenth-century rise of racial science and scientific racism. These narratives have also suggested that racial science and scientific racism were dealt a near-fatal blow in the early decades of the twentieth century when the German-American anthropologist Franz Boas and his students at Columbia University popularized a plural, relative, and non-hierarchical concept of culture (Gossett, 1963; Stocking, 1968; Stepan, 1982; Barkan, 1992; Lewis, 2014). For Boas and Boasian anthropologists, there were as many cultures as human societies, and cultures could not be hierarchically ranked on a scale of linear progression. Instead, each culture exhibited its own inviolable particularity, coherence, and rationality. Boasians used this democratized concept of culture to challenge racial science and scientific racism, which held that different races inherently possessed distinct and unchangeable human capacities, and that Western civilization formed the universal pinnacle of human potential (Boas, 1910, 1911, 1912; Benedict, 1940).

More recently, however, historians of the human sciences have examined the limits of this early-twentieth century liberal antiracism that was advanced through culturalism. As Lee Baker (2021, p. 137) writes, “Boas was an indefatigable crusader against scientists and policymakers who used their authority to argue that races and cultures were superior or inferior.” Against Social Darwinists and eugenicists, who argued that differences in the behavior and political-economic status of races reflected unequal biological inheritances which predestined white supremacy, Boas insisted on conceptually separating race and culture, and argued that biological race could not explain cultural differences or learned behaviors. Depending on the situation, Boasian anthropologists argued that race made little biological sense as it was used by scientific racists, or that race was merely biology and thus of little use in accounting for meaningful human differences (Pascoe, 1996). In the context of early-twentieth century scientific and policy debates around U.S. immigration quotas and miscegenation laws, the Boasian intervention was earnestly antiracist.

And yet, historians have also demonstrated how Boasian culturalists “articulated racism and contributed to the consolidation of whiteness” in their very efforts to be antiracist (Baker, 2021, p. 127; see also, Pascoe, 1996; Visweswaran, 1998, 2010; Baker, 2010; Anderson, 2019). For example, Boas maintained the scientific validity of race as a meaningful concept for designating differences among the “largest divisions of mankind,” while arguing that scientific racists misapplied the term (Boas, 1899, p. 294). Boas did not argue that races were equal or the same in their physical and mental capacities, but that scientific racists had not yet assembled convincing proof that observable differences were due to the innate superiority or inferiority of any race. Boas also routinely conflated whiteness with what it meant to be American in his scientific advocacy for liberal immigration policy and his assertions of immigrants’ capacity to assimilate in the United States. Finally, Boas mobilized the authority of science to advocate for racial amalgamation—specifically, “a continued increase of the amount of white blood in the negro community” (Boas, 1909, p. 848)—and to assuage concerns that this would have detrimental effects on either race. In light of these positions, Baker (2021, p. 140) describes Boasian culturalism as an exemplary form of “racist antiracism”—one that made significant contributions to both the fight against scientific racism and the emergence of new modes of racialization (Pascoe, 1996).

While conventional histories of the twentieth-century retreat of racial science position Boasian culturalism as the beginning of the end for scientific racism, they also suggested the repudiation of race in science remained unfinished until after World War II (Gossett, 1963; Stocking, 1968; Stepan, 1982; Barkan, 1992). In these accounts, 1950 marked the moment of a decisive break with race science: in the wake of world war and the Nazis’ genocidal eugenics, human and life scientists joined forces with liberal international organizations to expunge racism from science for good. At this time, the traditional, typological concept of race associated with nineteenth-century anthropology and twentieth-century eugenics was purportedly replaced by the more sophisticated, statistical concept of population associated with modern genetics (Haraway, 1989; Gannett, 2001, p. S480-S481; Reardon, 2005, pp. 20–22; Smocovitis, 2012). Old taxonomic and typological sciences of human difference—which used the concept of race to refer to essential, inherited, and unchanging distinctions between human groups understood as static racial types—were superseded by the “new, non-racial, populational, genetical science of human diversity,” called human population genetics (Stepan, 1982, p. 171).

But these narratives of postwar scientific redemption have also been critically re-examined by more recent histories and sociologies of the life sciences (Stoler, 1997, p. 185; Gil-Riaño, 2023, pp. 6–9). Scholars have illuminated the ways that race was not so much replaced or repudiated in postwar population genetics, but was rather redefined and repackaged in new technical idioms and research practices (Fujimura et al., 2010; Roberts, 2011; Bliss, 2012; Yudell, 2014; Nash, 2015; Nelson, 2016). Far from abandoning the race concept, population geneticists worked hard to conceptually reconfigure it in statistical, populational, and geographic terms precisely in order to preserve its scientific legitimacy. As Lisa Gannett demonstrates, establishing and promulgating the genetic race concept—which redefines races as populations that differ in gene allele or chromosomal type frequencies, with populations defined in turn as geographically delimited groups—was itself a principal aim and “product of the evolutionary synthesis” (2013, p. 250). So too was establishing the idea that the genetic concept of race represented a sharp break with typological conceptions of race: “the typological-population distinction ‘came to be employed instrumentally’ by scientists who in the 1950s and 1960s sought to distinguish their use of the concept of race from the legacy of the abuse of race by the Nazis in Europe and by segregationists and eugenicists in the United States” (Reardon, 2005, pp. 37–38).

As liberal postwar life scientists turned to population to render race politically neutral, many also adopted idioms of culture to continue designating human differences. The origins of cultural evolution research lay precisely in this turn. Just as some antiracist scientists strove to reform race in populational terms, others worked to harness culture to bypass race and the effects of racism (Lentin, 2005, p. 395). As a result, new forms of genetic and cognitive culturalism took shape, which borrowed heavily from Boasian culturalism’s armory of antiracist strategies. But as Michelle Brattain (2007, p. 1388) writes, “movements to dislodge racism are equally contingent, opportunistic, political, and grounded in the same social formations as racism itself.” Thus, a history of genetic and cognitive culturalisms contributes to the project of historicizing antiracism as well as the new forms of racialism that antiracist projects produce (Pascoe, 1996; Lentin, 2005; Brattain, 2007; Gil-Riaño, 2023).

## Race and intelligence in the U.S. civil rights era

In 1970, two events marked the emergence of cultural evolution. At the Anglo-Romanian Conference on Mathematics in the Archaeological and Historical Sciences in Romania, the Italian population geneticist Luigi Luca Cavalli-Sforza presented a paper in which he explored similarities between biological evolution and sociocultural change that might permit the mathematical formalization of the latter and the modeling of their interaction (Cavalli-Sforza, 1971). The same year, Cavalli-Sforza co-authored an article, “Intelligence and Race,” with the German-British geneticist Walter Bodmer for the *Scientific American* in response to high-profile pronouncements of scientific racism by the educational psychologist Arthur Jensen and physicist William Shockley (Bodmer & Cavalli-Sforza, 1970). Together, the two papers index the political and epistemological conditions in which population geneticists set themselves the task of mechanizing culture and quantifying genetic and cultural co-evolution.

Bodmer and Cavalli-Sforza’s aim in their article for the *Scientific American* was to intervene, in the name of genetic science and expertise, in a public controversy about the relationship between heredity, race, and intelligence. In the wake of the U.S. Supreme Court’s 1954 *Brown v. Board of Education* ruling, which mandated an end to racial segregation in public schools, opponents of integration mobilized eugenic arguments about the inherent inferiority of Black people’s intelligence in their attempts to fight desegregation (Roberts, 2011, pp. 46–47). A new cohort of scientific racists became increasingly prominent in the 1960s, as the Civil Rights movement shifted focus from securing equal rights and public accommodations toward positive efforts to improve conditions and services for racialized poor people (Brattain, 2007, p. 1410–1412; Yudell, 2014, pp. 175–177). Some of the most controversial parts of President Lyndon Johnson’s War on Poverty were Great Society programs such as Head Start, which provided educational resources for racially and socioeconomically “disadvantaged” children (Tucker, 1994, p. 181, 206–208). By the mid-1960s, “compensatory” educational programs—which variously offered school meals, remedial instruction, cultural enrichment activities, initiatives to raise children’s self-esteem, and comprehensive health examinations in under-resourced schools—became a principal target of critique for scientific racists and would-be eugenicists (Tucker, 1994; Brattain, 2007; Panofsky, 2014; Serpico, 2021).

In 1965, the Nobel Prize winning Stanford physicist William Shockley became a central figure in the race and intelligence debates when he delivered an address at a Nobel conference on “Genetics and the Future of Man.” Shockley claimed that one of the greatest threats to the future was the genetic deterioration of the human species (Tucker, 1994, pp. 183–185). He argued that the War on Poverty’s social programs were preventing evolutionary dynamics from eliminating genetically-inferior people; and that such programs, in fact, facilitated their increasing reproduction. As the U.S. Supreme Court ruled against segregation in education, housing, transportation, and public amenities, and the government introduced modest programs to improve the material conditions of racialized and impoverished Americans, eugenicists started to lose confidence that the people who they considered genetically inferior would disappear by force of evolutionary dynamics (Roberts, 2011, pp. 36–37). In this context, Shockley insisted alongside eugenicists and segregationists that governmental efforts to improve the educational conditions of Black children were useless and even harmful.

Shockley was “obsessed with public visibility…(and) became a one-man public relations firm and lobbyist for his eugenical views” (Tucker, 1994, p. 191). As early as 1964, leaders of the American Society of Human Genetics (ASHG) began considering the potential need to publicly address the growing role of genetics in social and political debates—prompted in large part by the prominence of appeals to genetics in arguments about racial differences of intelligence—and to contain Shockley specifically (Mitchell, 2017, pp. 429–431). Yet in 1964, and again in 1966, ASHG leaders decided to refrain from making any public statements about race. Prominent geneticists were wary of engaging with controversial issues and preoccupied with projecting a politically neutral public image for the discipline. There also remained disagreement among ASHG membership about the extent to which genetic science authorized the wholesale rejection of arguments like Shockley’s (Mitchell, 2017).

Not long after his Nobel address, Shockley was interviewed for a feature story about overpopulation and the proliferation of “inferior strains” in *U.S. News and World Report*, which *Stanford MD*, the School of Medicine’s alumni magazine, reprinted (Tucker, 1994, p. 184). Facing pressure from powerful donors, the faculty of Stanford’s Department of Genetics wrote a letter to the editor of *Stanford MD*, dismissing Shockley’s argument as a “pseudo-scientific justification for class and race prejudice,” which only warranted a response because of his standing as a Nobel Laureate on campus (Mitchell, 2017, pp. 429–431).^5^ But Shockley was undeterred. In 1966, he began making annual pleas to the National Academy of Sciences to study what he called the racial aspects of a heredity-poverty-crime nexus (Crow et al., 1967; Harris, 2023). His claim was that genetic disadvantages could explain high rates of poverty and crime in Black communities. And he suggested the government should switch sides in the War on Poverty and eliminate Black and poor people by erecting barriers to parenthood and conducting sterilization campaigns among supposedly inferior populations (Tucker, 1994). Meanwhile, Shockley was also active in recruiting other scientists to his cause.

Arthur Jensen, an educational psychologist based at UC Berkeley, spent the 1966-67 academic year at Stanford as a fellow at the Center for Advanced Study in the Behavioral Sciences. Jensen and Shockley began holding regular discussions, and Jensen’s research changed course while his arguments merged with Shockley’s. Jensen was invited by the editors of the *Harvard Educational Review* to write an extended lead article on heredity, race, and intelligence. And he “produced the most explosive article in the history of American psychology” (Tucker, 1994, p. 199). Over the course of 123-pages, Jensen argued that the answer to his titular question—“How Much Can We Boost IQ and Scholastic Achievement?” (1969)—was not very much. He wrote that, although “current thinking behind civil rights, fair employment, and equality of educational opportunity appeals to the fact that there is a disproportionate representation of different racial groups in the various levels of the educational, occupational, and socioeconomic hierarchy,” these differences could not be attributed to discrimination or the effects of slavery and segregation (1969, p. 79–80). Against the “dogma” of genetic equality that underwrote Civil Rights and the War on Poverty, Jensen argued that existing inequalities were best explained by the different genetic endowments of races.

Presenting himself as an expert in statistics, psychology, and genetics, Jensen declared that compensatory educational programs for “ethnic minorities and the economically poor” had resulted in “uniform failure” (1969, p. 3–4). Further, he wrote, they could not do otherwise because intelligence was genetically determined and practically fixed. While environmental deprivations might prevent a child from reaching their full potential, he claimed, no amount of educational enrichment could lift a child beyond the limits set by their genetics. For the genetically superior white population, Jensen recommended education including conceptual and abstract reasoning; but for genetically inferior Black and poor people, he argued, rote learning based on principles of operant conditioning would be more appropriate. He positioned his argument as ultimately more humane, warning of “a danger that current welfare policies, unaided by eugenic foresight, could lead to the genetic enslavement of a substantial segment of our population” (1969, p. 95).

Jensen’s article—and its arguments about Black genetic inferiority, which the *New York Times Magazine* dubbed “Jensenism”—triggered bitter public and scientific controversies (Tucker, 1994, p. 205). After three years of deferrals, the ASHG created a Social Issues Committee in 1967 to engage publicly with political questions of genetic science and society. However, upon establishing the committee, ASHG leaders once again demurred from making any statements about race, turning instead to questions of genetic screening and prenatal diagnosis. In 1969, the level of public attention surrounding Jensen’s article rekindled interest among ASHG leadership in publicly addressing the race and intelligence controversy. Yet, again, the committee decided that racial issues remained unsuited for any official ASHG action. Instead, the committee wrote to Walter Bodmer, who had signed the Stanford geneticists’ statement against Shockley, and asked him and Cavalli-Sforza to write an article detailing the problems with Jensen’s argument (Mitchell, 2017, pp. 439–440).

## The emergence of genetic culturalism

In their article for the *Scientific American*, Bodmer and Cavalli-Sforza (1970, p. 19) approached Jensen’s arguments with dispassionate neutrality in the name of objectivity and technical expertise: “We are geneticists who are interested in the study of the interaction between heredity and environment. Our aim is to review, mainly for the nongeneticist, the meaning of race and I.Q. and the approaches to determining the extent to which I.Q. is inherited.” Such a review, they claimed, could form a basis for the evaluation of claims about the genetic determination of measured IQ differences between races. Bodmer and Cavalli-Sforza devoted much of their essay to explaining the ways that population geneticists operationally defined intelligence, race, and inheritance, as well as the proper ways of interpretating relative, probabilistic, and statistical measures of genetic markers in populations. They explained that “complex behavioral traits such as intelligence” are influenced by the combined action of many genes, resulting in a level of complexity that eluded extant statistical tools and the scope of genetic explanations (Bodmer & Cavalli-Sforza, 1970, p. 19). They also drew attention to the influence that environment has on gene expression, especially in the case of complex traits, adding another layer of technical obscurity to the matter.

Their stance was accommodating: “currently available data are inadequate to resolve this question in either direction” (Bodmer & Cavalli-Sforza, 1970, p. 29). They did not foreclose the possibility that genetics contributed to racial differences in IQ. They did not reject or denounce the values that Jensen’s and Shockley’s scientific racism articulated. Instead, they insisted that, given the present state of the science, it was impossible to know and no conclusion could be drawn. They suggested that to argue, as Jensen and Shockley had, that “biological inheritance of the simplest kind entirely determines I.Q,” was to betray statistical illiteracy and scientific naivety (Bodmer & Cavalli-Sforza, 1970, p. 23). Bodmer and Cavalli-Sforza’s aim was to delegitimize racist claims by technically adjudicating good genetic and statistical science while maintaining a positivist commitment to value-free technical evaluation. Culture provided an epistemic resource for this liberal antiracism, allowing for a conceptual deflection of race as an appropriate analytic category. Racial explanations of measured IQ differences had employed the wrong tools for the job; rather than race, such differences had to be understood with reference to cultural inheritance.

Although no conclusions about the genetics of intelligence could be drawn, Bodmer and Cavalli-Sforza argued that environmental factors—including “both the lower socioeconomic status of U.S. blacks and a cultural inheritance dating back to slavery”—could explain the differences in measured IQ between the two populations (Bodmer & Cavalli-Sforza, 1970, p. 28–29). Appealing to the logic of experimental control, they argued that the question of a possible genetic basis for differences in measures of intelligence would be impossible to answer scientifically until the “environmental differences” between Black and white Americans were dramatically reduced:

It is difficult to see, however, how the status of blacks and whites can be compared. The very existence of racial stratification correlated with a relative socioeconomic deprivation makes this comparison suspect. Black schools are well known to be generally less adequate than white schools, so that equal numbers of schooling certainly do not mean equal educational attainment. (…) No amount of money can buy a black person’s way into a privileged upper-class white community, or buy off more than 200 years of accumulated racial prejudice on the part of the whites, or reconstitute the disrupted black family, in part culturally inherited from the days of slavery. It is impossible to accept the idea that matching for status provides an adequate, or even a substantial, control over the most important environmental differences between blacks and whites (Bodmer & Cavalli-Sforza, 1970, p. 27).

For Bodmer and Cavalli-Sforza, the controversy surrounding race and IQ raised questions about the emergence of human differences that could not be explained by genetics. Given their commitment to the idea that all human beings shared a universal evolutionary history, they argued that explaining the differences in measured IQ among Black and white Americans would require accounting for the specific inheritances of slavery, segregation, and racial prejudice. For these geneticists, explaining inheritance on this short of a timespan raised the question of cultural inheritance and differentiation, which they posited was capable of introducing variation more quickly than biological evolution. In the *Scientific American*, Bodmer and Cavalli-Sforza argued that disentangling the relative contributions of biological and cultural factors which led to the formation of complex behavioral characteristics such as intelligence remained beyond the reach of genetic science. But Cavalli-Sforza was working on it.

At the conference in Romania, Cavalli-Sforza (1971) presented a paper titled, “Similarities and Dissimilarities of Sociocultural and Biological Evolution.” He opened with the claim that the modern mathematical theory of biological evolution, whose “foundations were laid in the twenties by three people, R.A. Fisher, J.B.S. Haldane, and S. Wright,” was the most important development in the life sciences to date (1971, p. 535). He emphasized how remarkable it was that a complex process like biological evolution could be examined quantitatively. What had made this feat of quantification possible—what Cavalli-Sforza argued had been the key to success—was the “isolation of some fundamental ‘factors’ of evolution which are easily quantified” (1971, p. 535). Building a modern evolutionary science of culture, he argued, would require the identification of an equivalent to the Mendelian mechanism of genetic transmission in the domain of sociocultural change.

Cavalli-Sforza set out to compare the major factors of biological evolution—mutation, selection, migration, and drift—with what seemed to him to be equivalent factors of sociocultural change. He reported being “greatly surprised by the existence of considerable similarities” between the two kinds of evolution (1971, p. 535). In biological evolution, mutations drove hereditary variation; in sociocultural evolution, he posited “close parallels between mutation and the processes giving origin to new ideas, *invention*” (1971, p. 536). He argued that natural selection was just as applicable to new ideas as it was to genetic mutations. And, as a formal equivalent of fitness, he proposed calculating the probability that a given innovation or new idea would be accepted by other individuals to whom it was transmitted. Since migration drove the spread of ideas even more easily than that of genes, he concluded that it must play an equally important role in the two types of evolution. Similarly, he reasoned, random genetic drift very likely had formal equivalents in cultural evolution, since “in the spread of any innovation, chance must play a role” (1971, p. 538).

Cavalli-Sforza also argued that there were important differences between biological and cultural transmission. Biological inheritance operating through the Mendelian mechanism of genetic transmission is strict: genes are passed from parents to offspring only. Conversely, cultural transmission is not: parent to child transmission still plays some role, “but not such a rigid one, and in addition, a very large fraction of our knowledge derives from interactions between teacher and pupil, sib and sib, friend and friend. Indirect transmission through books, mass media, and so on, takes an ever-greater share” (1971, pp. 536–537). Cavalli-Sforza argued that, in order to build a quantitative theory of cultural evolution, these distinctly cultural mechanisms of transmission would have to be mathematically formalized and modeled. This was also the only hope for scientifically disentangling the relative contributions of biological and cultural factors to complex behavioral characteristics such as intelligence.

Cavalli-Sforza’s interest in culture had been inspired by fieldwork “expeditions” that he conducted in Central Africa in the late 1960s. “I started trying to understand something about cultural evolution,” he told his biographers, “when I saw how different the Pygmy way of life was from that of others” (quoted in Stone & Lurquin, 2005, p. 77; Cavalli-Sforza, 2000, p. xi). His first expedition was in 1966, and he returned every winter until 1971. The motivation driving Cavalli-Sforza’s fieldwork was the “desire to study one of the few remaining groups of hunter-gatherers left in the world” (1986, p. 1). Cavalli-Sforza was fascinated by the fact that “for 99% of their history, humans lived as hunter-gatherers,” until about 10,000 years ago when techniques of plant and animal breeding for food production altered the course of human history—or, at least, the history of most of the species. For “salvage biologists” like Cavalli-Sforza, social and geographic “isolates” such as Central African Pygmies provided geneticists with an opportunity to travel 10,000 years back in time. As Joanna Radin (2017, p. 111) writes, “to Cavalli-Sforza, primitive communities were not necessarily people without history but people who *were* history.” In Africa, Cavalli-Sforza sought prehistory in the present.

Cavalli-Sforza’s expeditions were organized around the collection of blood. As he described in the introduction to *African Pygmies*, the objective of his first field trip “was to locate Pygmies, since their whereabouts were only vaguely known, and then to convince them to give us blood samples” (1986, p. 3). Cavalli-Sforza and his team were hosted at the La Maboké Station Expérimentale—an outpost of the Musée national d’histoire naturelle in the Central African Republic—and he initially worked with local plantation owners and farmers to make contact with Pygmy villages. Among the groups living near the station, however, he became suspected of *likundu*. His team started venturing to more remote locations, “where there was no chance that the rumor might have spread,” and where his team offered Pygmy communities gifts such as salt, soap, cigarettes, and basic medical treatment in return for their blood (1986, p. 3–4). Collection was systematic: “we bled all members of a camp except for small children” (1986, p. 4). During his first trip, Cavalli-Sforza “worked out a routine for examination, blood collection storage, and shipping,” which guaranteed that blood samples would arrive “in European laboratories 7 days after collection, if not sooner” (1986, p. 4, p. 6).

Cavalli-Sforza wanted Pygmy blood to analyze for genetic markers. His aim was to study the structure of populations, considering Pygmies to be exemplars of human evolution before the advent of farming. By the time Cavalli-Sforza made his first expedition to Africa, he had several lines of ongoing research in Europe that analyzed gene frequencies of blood types and DNA polymorphisms to calculate the “genetic distance” between populations (Edwards, 2021, pp. 90–91). After gaining access to parochial church records of all births, marriages, and deaths in the Italian Parma Valley since the Council of Trent (1563), he took blood samples across the region, compiled genetic and demographic data, and constructed evolutionary trees organizing populations according to their genetic distances (Cavalli-Sforza, 1966, 2000, p. x; Edwards, 2021, pp. 88–89). He also collected blood in Puglia and Sardinia and collaborated with archaeologists to theorize the spread of agricultural practices from the Middle East to Europe (Stone & Lurquin, 2005, pp. 86–87). Cavalli-Sforza’s expeditions to Africa constituted an effort to extend the geographic range of this blood sampling and to make use of the Pygmies’ recent evolutionary isolation. “We can simplify the process” of reconstructing human evolution, he wrote, “by concentrating most of our studies to indigenous people, when it is possible to recognize them and differentiate them from recent immigrants to a region” (2000, p. 18).

Cavalli-Sforza considered these different lines of research to be parts of a unified project that he had begun conceiving while studying bacterial genetics in R. A. Fisher’s laboratory at the University of Cambridge in the late 1940s. In Fisher’s lab, a “place saturated with mathematical theorizing,” Cavalli-Sforza started thinking about how genetics could be used for “the reconstruction of where human populations originated and the paths by which they spread throughout the world” (Cavalli-Sforza, 1991, p. 104). As Cavalli-Sforza moved from bacterial genetics to human population genetics, he became convinced that human migration and genetic drift had played pivotal roles in the peopling of Europe. Studying genetic distances between populations on different continents and developing new methods for building evolutionary trees convinced him that all humans had a common origin in Africa, and that the spread of populations from there could be reconstructed on the basis of genetic data drawn from living populations (Cavalli-Sforza, 2000, p. 33). By the late 1960s, Cavalli-Sforza’s different lines of research were all pointing to the massive changes introduced into the trajectory of human evolution by the emergence and spread of culture.

In 1968, Cavalli-Sforza took a sabbatical from the University of Pavia in Italy, where he was professor of genetics, the Instituto di Genetica, and the Pavia Section of the Laboratorio Internazionale di Genetica e Biofisica, where he was the director. He spent the 1968-69 academic year at Stanford University, where he and Walter Bodmer worked toward completing their manuscript of *The Genetics of Human Populations* (1971). Cavalli-Sforza later told his biographers that his time at Stanford had solidified his interest in culture. Although his fieldwork expeditions and efforts to reconstruct prehistorical human movements had provided the initial prompts for him to consider culture more deeply, his experience in California had offered him another perspective on the concept. As he explained, it was at Stanford that he realized “the concept of cultural learning was a valid weapon against racist arguments that differences between people (for example, different IQ scores among ethnic groups) were due to biologically determined ‘racial’ differences” (Stone & Lurquin, 2005, p. 86). Cavalli-Sforza considered culture a useful scientific object precisely because it could be used to legitimize liberal values on the grounds of evolutionary science.

In 1971, Cavalli-Sforza relocated to Stanford and started a professorship in the Department of Genetics. During his first year, he gave a talk on campus to a group of mathematical biologists that was aimed at identifying formal analogies between biological and cultural evolution which could then be mathematically modeled. One of the biologists who attended Cavalli-Sforza’s talk had also started a professorship at Stanford in 1971: Marcus Feldman, who had completed his PhD at Stanford under Bodmer’s supervision in 1969. Feldman later recalled waiting around after Cavalli-Sforza’s talk to ask him a few technical questions; once the audience dispersed, they “sat down right there and solved a few of the mathematical problems—that’s how it all began” (Feldman quoted in Stone & Lurquin, 2005, p. 97). Soon, Cavalli-Sforza and Feldman were meeting “three or more nights a week at Cavalli’s house, carrying out mathematical computations and fleshing out ideas about cultural transmission” (Feldman quoted in Stone & Lurquin, 2005, p. 97). Their first co-authored papers appeared in the spring of 1973.

Over the course of the next decade, Cavalli-Sforza and Feldman published more than two dozen articles on cultural transmission mechanisms and cultural evolution, culminating in their monograph, *Cultural Transmission and Evolution: A Quantitative Approach* (1981). The book laid out, over almost 400 pages, a mathematical theory of cultural change with each chapter devoted to modeling different cultural transmission mechanisms. Cavalli-Sforza and Feldman (1981, p. 7) operationalized culture as referring to “traits that are learned by any process of nongenetic transmission, whether by imprinting, conditioning, observation, imitation, or as a result of direct teaching.” This conceptual formalization of culture as those “aspects of ‘thought, speech, action (meaning behavior), and artifacts’ which can be learned and transmitted” configured culture as a property that things can obtain by virtue of being transmitted (Cavalli-Sforza & Feldman 1981, p. 10). In addition to its amenability to mathematical modeling, this definition had the benefit of defining culture as an epistemic object that could in fact only be understood with the theoretical and methodological tools of population genetics. Culture designated precisely that which had been nongenetically transmitted and subject to natural selection.

In the preface to *Cultural Transmission and Evolution*, Cavalli-Sforza and Feldman (1981, p. vii) wrote that they were in the process of writing a second volume that would apply their mathematical theory and models to the specific problem of “individual, inherited differences in learning ability.” This second book never appeared, and the relation between race and culture was indefinitely deferred. In 1991, Cavalli-Sforza announced the launch of the Human Genome Diversity Project, which was another attempt to extend the reach of his efforts to salvage cultural and genetic data for an evolutionary science of universal human origins (Cavalli-Sforza et al., 1991; M’charek, 2005; Reardon, 2005). Cavalli-Sforza and Feldman stopped publishing on the topic of cultural transmission and evolution in the mid-1980s, but they set the terms that continued to animate debate among cultural evolutionists into the twenty-first century.

## A second system of inheritance

According to Peter Richerson (personal communication (interview), June 18, 2019), the “genesis” of his work on cultural evolution was teaching in the early 1970s. In 1971, he joined the faculty of the new Division of Environmental Studies at UC Davis. That year, one of the division’s founders, James McEvoy, asked Richerson to co-teach a course called Principles of Human Ecology. Trained as a sociologist, McEvoy “wanted to co-teach the course with a natural scientist” to explore the division’s promise of interdisciplinarity. But co-teaching across the social and natural sciences raised conceptual questions. “We decided to make adaptation one of the main themes,” Richerson explained. “I was an ecologist by training, so I thought I knew how adaptations came about. But since this was *human* ecology, one of the obvious things was that humans learn a lot from each other—so, culture is an important phenomenon.” Thinking within the thematic frame of their course, Richerson recalled being hung up on seemingly basic questions: “What *is* a cultural adaptation? How would cultural adaptations come about?”

Richerson recounted searching across several fields for answers, turning first to human ecologists and then to anthropologists, but finding their work on the evolution of culture to be “scanty in the extreme.” His “chance discovery” of the American psychologist Donald Campbell’s efforts to reinvigorate social evolutionism in light of evolutionary genetics—presented first at a conference on the uses of evolutionary theory for social sciences at Northwestern University in 1961, and then in his Presidential Address at the 1975 annual meeting of the American Psychological Association—provided Richerson with crucial encouragement (Boyd & Richerson, 1985, p. vii; Phillips, 1971; Campbell, 1976). It was essentially a programmatic essay, Richerson explained. Campbell simply argued that “you could study cultural evolution like biologists study genetic evolution—it’d be somewhat similar and somewhat different, and that was about the extent of it” (Richerson, personal communication (interview), June 18, 2019). Yet, however provisional, Campbell’s proposal struck Richerson as an “intelligent middle ground between complete genetic determinism and complete cultural determinism” (Boyd & Richerson, 1976, p. 254).

In his Presidential Address, Campbell (1976, p. 177) characterized nineteenth and early twentieth century social evolutionists like Herbert Spencer as “too much despised.” The biggest issue with early social evolutionists, he claimed, was that they had paid “no attention to natural selection analogues in the process of social evolution” (1976, p. 171). Their studies were overly descriptive and did not attend to the “mechanisms that would make an adaptive evolutionary progress possible” (1976, p. 171). This limitation was historical rather than the fault of individuals, he reasoned apologetically, as it was only in the mid-twentieth century that modern evolutionary biologists had laid the foundation for a rigorous study of social evolution with modern evolutionary genetics—and, more recently, with sociobiology. The latter in particular had elevated the importance of understanding cultural inheritance, Campbell claimed—describing Wilson’s *Sociobiology* as “magnificent” (1976, p. 179)—since the extreme sociality of humans cannot be predicted or explained by genetic competition and the individual selfishness for which it selects. For Campbell, culture curbed nature, allowing humans to optimize social coordination in spite of natural selection. The object of this reconfigured social evolutionism, he argued, should be the mechanisms of sociocultural evolution that allowed humans to transcend their selfish nature. Campbell suggested that, just as culture curbed nature, so scientific consideration of culture could curb scientific racism.

In 1971, the same year that Richerson joined the faculty in the Division of Environmental Studies at UC Davis, Robert Boyd started his PhD there. In 1974, after Boyd advanced to doctoral candidacy, he and Richerson co-taught an introductory environmental studies course for undergraduates (Boyd & Richerson, 1985, p. vii; Richerson & Boyd, 2005, p. vii). While teaching together, Richerson began sharing with Boyd what he had been learning about cultural evolution. They started holding weekly meetings to work on developing a theory of cultural evolution. “Rob had no training in evolutionary biology,” Richerson said (personal communication (interview), June 18, 2019). “Basically, he was a physicist as an undergraduate, so that’s where he got his applied math chops. That was the division of labor between us, he was the math guy. We started thinking about what sort of models we could make.”

Boyd and Richerson published their first paper on cultural evolution in 1976, followed by a second in 1978. In both articles, Boyd and Richerson praised Campbell for identifying the pertinence of the genetic reinterpretation of Darwinian selection for evolutionary studies of culture. They expressed agreement with Campbell that human behavior was shaped by selection acting on both culture and genes. But they departed from Campbell’s sociobiological interpretation of a conflict between cultural and genetic evolution, arguing that more theoretical and empirical research was needed to warrant such a conclusion: “given our primitive level of understanding of cultural evolutionary mechanisms, it is premature to attempt to explain the broad features of human society.” Instead of assuming a conflictual relation between genetic and cultural evolution, as sociobiologists did, Boyd and Richerson (1976, pp. 254–255) aimed to make the nature of this relationship their object of study: “Our own approach has been to consider the simplest possible mathematical models of the interaction of culture and genes in the hope of obtaining at least a clear, if rudimentary, picture of the mechanisms involved.” Once more specific mechanisms had been modeled, these could be used to “develop a rigorous theory of human behavior on a par with population genetics in evolutionary biology” (1976, p. 260–261).

Like Cavalli-Sforza, Boyd and Richerson positioned their project as fulfilling the promise of the modern synthesis of natural selection and genetics that Fisher, Haldane, and Wright had initiated in the 1930s. Despite successful applications of Neo-Darwinian theory to a growing range of complex ecological phenomena, Boyd and Richerson (1978, p. 128) wrote, human “behavioral peculiarities caused by our capacity for culture” had continued to elude evolutionary explanation. In short, they argued that cultural processes created human behaviors which could not be explained by genetic evolution alone (Richerson & Boyd, 2005). The problem confronting evolutionary biologists in their view was that “much of human behavior is acquired through cultural mechanisms rather than determined by genetic inheritance” (1978, p. 128). They postulated that culture comprises a “second system of inheritance” through which information affecting behavior is transmitted by distinct mechanisms. In contrast to complete genetic reductionists who claimed behavior could be predicted by an organism’s genotype, and epigeneticists who predicted behavior on the basis of interactions of a genotype and its environment, Boyd and Richerson argued that to predict the behavior of a cultural organism, “one must know its genotype, its environment, and its ‘culture-type’” (1978, p. 128). This was, in short, the ambition of their “dual inheritance theory.”

Boyd and Richerson’s theoretical proposal rested on defining culture as a system of inheritance that interacts with genetic transmission while remaining distinct from it. They insisted that “culture is not inherited by the same mechanism as genes,” and that the differences were in fact numerous: the rates and temporalities of genetic and cultural transmission differ considerably; the contributions from parents to offspring are not automatically equal in the case of cultural transmission; and further, culture is often transmitted among totally unrelated individuals (1978, p. 134). Boyd and Richerson’s first aim was to create mathematical models of the interactions between the genetic and cultural systems of inheritance. They described their first model as “formally identical with a two-person, non-zero-sum game played between culture and genes” (1976, p. 258, 1978, p. 129). Using a Nash equilibrium analysis of this game between culture and genes, Boyd and Richerson purported to deduce “the broad evolutionary features of the dual inheritance system” (1978, p. 131). Humans behaved, they claimed, as though the genetic and cultural inheritance systems were each strategizing individuals playing a game in which the stakes are fitnesses. For Boyd and Richerson, the most important conclusion of the simple dual inheritance model was its purported demonstration that cultural transmission could lead to genetically advantageous behaviors that could not be produced by genes or genetic evolution. This demonstrated the necessity, they claimed, of studying culture as a distinct system of inheritance.

In both papers, Boyd and Richerson lamented the fact that reactions against Social Darwinism had led many social scientists to neglect the modern genetic reinterpretation of natural selection. This neglect, however, established an epistemic opening that they designed their program to fill. They did not address the racist history and politics of Social Darwinism, but rather repeated Campbell’s critique that it lacked sufficient scientific rigor. “Very little of it,” they wrote, “is properly within the Darwinian tradition” (1978, p. 130). Like Bodmer and Cavalli-Sforza’s intervention in the race and IQ debates, Boyd and Richerson’s stance toward scientifically racist social evolutionism was that it represented an ideological and unsophisticated corruption of science. For Boyd and Richerson, the modern synthesis had successfully refuted racist uses of evolutionary theory—or at least, circumvented and precluded them. They declared confidently that their approach afforded “no ‘eugenic’ possibility in the sense that the models do not permit culture as a whole to reduce individual genetic fitness in order to increase cultural fitness” (1976, p. 260). Natural selection acting on genes optimized genetic fitness by controlling the evolved capacity for culture, the positive effects of which had to exceed the negative ones for the very capacity to persist in a population.

After defending his PhD in 1975, Boyd had begun working as a consultant for the newly created California State Energy Commission doing electricity demand forecasting. As he wanted to return to academia, he and Richerson decided to use Richerson’s sabbatical in 1977 to start working on a book-length treatment of dual inheritance theory. They asked E. O. Wilson if they could spend the year with his research group at Harvard, but “he said no—that his lab was full” (Richerson, personal communication (interview), June 18, 2019). “(Wilson’s) take on culture was radically different from ours,” Richerson added. “Our idea, exemplified in that game theory paper, was that culture and genes were sort of co-equal evolutionary processes, and one would influence the other, but genes were not overwhelmingly dominant. That was radically different from Wilson.” In Richerson’s view, the “model that leads to (Wilson’s) thousand-year rule is a mechanism of phenotypic flexibility; there isn’t any real cultural transmission in it” (personal communication (interview), June 18, 2019).

Not long after Wilson turned them away, Boyd and Richerson learned that Cavalli-Sforza and Feldman were teaching a seminar at Stanford on approaches to mathematically modeling cultural transmission, which Cavalli-Sforza and Feldman allowed them to sit in on. “So, (Boyd) and I went down to Berkeley and rented a house,” Richerson recalled (personal communication (interview), June 18, 2019). “We drove across the Bay once a week to attend their seminar, and started to work on what became the eighty-five book.” *Culture and the Evolutionary Process* (1985) would become the founding text of the California school of cultural evolution and the urtext of Boyd and Richerson’s dual inheritance theory. Dual inheritance theory adopted the general terms set by Cavalli-Sforza and Feldman for a science of cultural evolution, aiming to model cultural transmission mechanisms and quantify genetic and cultural co-evolution. But it departed from their thinking by arguing that cultural evolution must be adaptive and beneficial in terms of biological fitness (Boyd & Richerson, 1985, p. 14).

Boyd and Richerson also demonstrated a greater sociological self-consciousness than Cavalli-Sforza and Feldman, carefully distinguishing at length and in technical detail how their conceptualization of culture differed from those of sociobiologists, social scientists, and other evolutionary biologists. Aware of American cultural anthropologists’ postcolonial and postmodern critiques of concepts of culture, Boyd and Richerson began incorporating a history of anthropology’s betrayal of objective science into their programmatic texts to legitimize their claims to authority over culture as an epistemic object. By the late 1980s and 1990s, their work was wholly focused on mathematically modeling transmission mechanisms in the name of politically neutral, positivist evolutionary science. Liberal antiracism and evolutionary humanism remained present only at the level of assumptions. In Boyd and Richerson’s publications, it no longer appeared as a motivating force of research. Instead, they argued, the excesses of both postmodern anthropologists and genetic determinists necessitated a more sober scientific middle ground.

When I asked Richerson if he ever worried about cultural evolution being taken up by scientific racists, he replied,

“It’s something we worry about. People that have that turn of mind turn to genes. But you could make a whole scientific racist theory out of cultural evolution. Cultural evolution is susceptible to exactly the same abuse that genetic evolution is in the hands of someone who wants to use it maliciously. I don’t think anyone’s done that. Certainly, none of the influential figures in the field have gone down that road. Rob’s and my personal politics are liberal. Feldman’s had a war on heritability of IQ for years and years, so he’s on the side of the angels. I don’t have any idea what Cavalli-Sforza’s politics were” (personal communication (interview), June 19, 2019).

## The cognitive turn in Cultural Evolution

In 2014, Daniel Dennett organized a workshop on cultural evolution at the Santa Fe Institute in New Mexico to address “misunderstandings” that had arisen in recent years between different schools of cultural evolution, and to “find common ground and resolve differences among some of the leading theorists and experimentalists” (2017, p. 2). The meeting’s participants reflected the major divisions within the field as it stood in the first decade of the twenty-first century. Dennett, accompanied by Susan Blackmore, participated as representatives of memetics, an approach to cultural evolution that grew out of sociobiology. Peter Richerson and Robert Boyd, accompanied by a former student and frequent collaborator, Joseph Henrich, represented the California school of cultural evolution. The French cognitive scientists Dan Sperber, Nicolas Claidière, and Olivier Morin represented the Paris school. Finally, the philosophers of biology Peter Godfrey Smith and Kim Sterelny participated as long-term observers of the debates between these traditions.

Memetics was first proposed by Richard Dawkins in the final chapter of *The Selfish Gene* (1976). Seemingly aware that he had painted a dismal portrait of life, Dawkins ended on a kind of anthropocentric optimism: “We (humans), alone on earth, can rebel against the tyranny of the selfish replicators,” arguing that what made humans capable of overcoming their selfish genes was culture (1976, p. 201). He also claimed that “cultural transmission is analogous to genetic transmission” in giving rise to a form of evolution, and argued that understanding human evolution in its entirety required grasping both of these processes (1976, p. 189). For Dawkins, genes derived their importance from being the replicating entities whose differential survival was the stuff of biological evolution. But Dawkins agreed with other cultural evolutionists that, in the case of human evolution, genetics had a supplement that had yet to be theorized. To understand cultural evolution, he wrote, “we need a name for the new replicator, a noun that conveys the idea of a unit of cultural transmission.” He offered *meme*. Just as genes selfishly propagated themselves for their own sake, “so memes propagate themselves in the meme pool by leaping from brain to brain via a process which, in the broad sense, can be called imitation” (1976, p. 192). Blackmore (1999) and Dennett (1995) adopted Dawkins’s proposal, developing it under the rubric of memetics in the 1990s.

In *Culture and the Evolutionary Process* (1985), Boyd and Richerson joined sociobiologists in ascribing special importance to imitation as the behavioral hinge of cultural transmission, writing “by ‘culture’ we mean the transmission from one generation to the next, *via teaching and imitation*, of knowledge, values, and other factors that influence behavior” (1985, p. 2, emphasis added). Individual learning, they wrote, can be costly and error prone: individuals may fail to learn locally adaptive behaviors, or alternatively, retain maladaptive ones only because they have been reinforced by chance. When the costs of errors are high, selection would favor shortcuts to learning. “Cultural inheritance is adaptive because it is such a shortcut,” they claimed. “If the locally adaptive behavior is more common than other behaviors, imitation provides an inexpensive way to acquire it” (1985, pp. 14–15).

The Paris school took shape more recently than other prominent approaches to cultural evolution, consolidating only in the early twenty-first century around the French cognitive scientist, Dan Sperber, and his students at the Institut Jean Nicod in Paris. While members of the Paris school have agreed with the California school, as well as sociobiologists and memeticists, that culture must be conceptually reconfigured and examined in terms of cultural transmission mechanisms to bring it within the framework of evolutionary science, they have strongly contested the California school’s claims about how such mechanisms work. Drawing on cognitive science and evolutionary psychology, members of the Paris School rejected the California School’s formalization of imitation as the behavioral substrate of cultural transmission. Instead, on the basis of linguistic and psychological laboratory experiments with humans and nonhuman primates, they argued that the forms of cognition involved in cultural transmission were more complex than models of imitation and copying behaviors admit. Examining the “cognitive mechanisms producing” cultural transmission, they insisted, reveals not imitation but “nonrandom (i.e. biased) transformations” at the heart of cultural transmission processes (Scott-Phillips et al., 2018, p. 162).

Sperber’s training began at the Sorbonne where he worked with the French anthropologist Georges Balandier. In the early 1960s, he spent two years at Oxford, studying with the structural anthropologist Rodney Needham. When Sperber returned to Paris in 1965, he secured a position with the *C*entre National de la Recherche Scientifique (CNRS), first at the Laboratoire d’etudes africaines and then at the Laboratoire d’ethnologie de Nanterre. As the position came with a lifetime appointment at the CNRS, he did not complete a doctorate (Dan Sperber, personal communication (interview), April 13, 2019). In the course of the 1970s, Sperber gained notoriety for his trenchant critiques of structural and symbolic anthropology (1973, 1975, 1985), becoming the “most prominent young critic of Claude Lévi-Strauss in Paris” (Carlo Severi, personal communication (interview), February 28, 2020). By the 1980s, Sperber’s critical appraisals of social and cultural anthropology led him to collaborate with linguists and cognitive psychologists who were interested in studying human culture from evolutionary and experimental perspectives (Sperber, 1996).

Sperber recalled that his interest in the field of cultural evolution was piqued in the mid-1980s when Jacques Mehler, the founder of the journal *Cognition*, asked him to review a paper by Leda Cosmides and John Tooby (personal communication (interview), April 13, 2019). In the late 1980s and early 1990s, Cosmides and Tooby published a series of articles outlining their program of evolutionary psychology. They praised sociobiologists in particular for prompting scientific progress in the evolutionary study of human behavior, but criticized evolutionary biologists for “overlook(ing) a crucial link in the causal chain from evolution to behavior: the level of innate psychological mechanisms” (Cosmides & Tooby, 1987, p. 277). When population geneticists and evolutionary biologists operationally defined cultural transmission mechanisms in terms of imitation and copying behaviors, Cosmides and Tooby argued, they mistook evolutionary mechanisms for the expressions of those mechanisms. The problem “consisted of attempting to apply evolutionary theory directly to the level of manifest behavior, rather than using it as a heuristic guide for the discovery of innate psychological mechanisms” (Cosmides & Tooby, 1987, pp. 278–279).

In Cosmides and Tooby’s account, evolutionary forces acted on the brain, which lead to adaptive behavior, rather than acting directly on behavior. Without attending to the level of psychological or cognitive mechanisms, they wrote, cultural evolutionists had offered “nothing more than post hoc compilations of correspondences between behavior and loosely reinterpreted evolutionary theory” (Cosmides & Tooby, 1987, p. 282). They positioned evolutionary psychology as the “missing link” that would unify evolutionary biology and the cognitive sciences (Cosmides & Tooby, 1987; Cosmides et al., 1992).

Sperber adopted a similar position in the 1990s, when he outlined his vision for a naturalistic science of culture. Before he began intervening in the field of cultural evolution, he worked between anthropology and psychology, advocating for a disciplinary separation of ethnography—to be tasked with understanding particular varieties of human cultural experience—and “theoretical anthropology,” which would explain the cognitive capacity for cultural variability in the first place (Sperber, 1996). A truly scientific anthropology, Sperber argued, had to abandon the interpretive methods of ethnography and deepen its engagement with the mind and brain sciences. Sperber explained that this conviction had been inspired, “in a strange way,” by Claude Lévi-Strauss’s ideas about how myths influence one another: “there is a kind of chain, or web, or network of transmission over time” (Sperber, personal communication (interview), April 13, 2019). “The point,” he explained, “is that cultural ideas contribute to their own success, they don’t fulfill some kind of biological function for humans. Cultural items don’t have to serve the populations in which they occur, they have to serve their own propagation” (Sperber, personal communication (interview), April 13, 2019).

In *Explaining Culture: A Naturalistic Approach* (1996, p. 3), Sperber outlined a research program that he called “the epidemiology of representations.” Inspired by developments in the cognitive sciences, Sperber aimed to reconceptualize human culture in the naturalistic terms of cognitive psychology. Sperber argued that the social sciences had postulated an “ontological autonomy of culture” in order to “insulate anthropology from both biology and psychology;” as a result, anthropology lacked theoretical concepts that could be incorporated into evolutionary frameworks (1996, p. 10). Sperber thus set himself the task of naturalizing culture and anthropology by reconceptualizing “the whole ontology of the social sciences,” writing:

Let us start as simple as possible. Cultural things are, in part, made of bodily movements of individuals and of environmental changes resulting from these movements. For instance, people are beating drums, or erecting a building, or slaughtering an animal. The material character of these phenomena is, so far, unproblematic. But we must go further. Is it a musical exercise, a drummed message, or a ritual? Is it a house, a shop, or a temple? Is it butchery, or sacrifice? In order to answer, one must, one way or another, take into account the representations involved in these behaviours. Whatever one’s theoretical or methodological framework, representations play an essential role in defining cultural phenomena. But what are representations made of? Let us note, to begin with, that two types of representations are involved: mental representations and public representations. (…) The question is: why do some representations propagate, either generally or in specific contexts? (Sperber, 1996, pp. 24–25)

Sperber wrote that the epidemiology of representations would account for “the material interactions of brains, organisms and environment which explains the distribution of (mental and public) representations” (1996, p. 26). With the tools of the cognitive sciences, mental representations could be theoretically operationalized as “brain states described in functional terms” (Sperber, 1996, pp. 24–26). As a result, it was scientifically possible to treat mental representations (such as ideas) and public representations (such as speech acts) as materially commensurable things whose relays could be tracked: “What I call ‘chains’ are, of course, quite complex, and generally look like webs, networks, or lattices. Still, they are made of only two types of links: from the mental to the public and from the public to the mental” (Sperber, 1996, p. 26). “To explain cultural representations,” in Sperber’s estimation, was ultimately “to explain why some representations are widely shared” (1996, p. 82). Culture designated those representations which are transmitted widely enough to be stabilized across a human population (Sperber & Hirschfeld, 2004).

In *Explaining Culture*, Sperber lamented that all existing approaches to the study of cultural transmission “share a crucial defect: they take the basic process of cultural transmission to be one of replication, and consider alterations in transmission as accidents” (1996, p. 82). While this view was primarily grounded in a “biological analogy,” it was shared by social scientists in a philosophically idealistic fashion. From a materialist and cognitive perspective, cultural transmission in fact involved the patterned transformation of representations. The cognitive psychology of cultural transmission was far more complex than biologists had granted—a claim that became the foundational intervention of the Paris school of cultural evolution and the primary axis of disagreement between Parisians and Californians.

Sperber and his collaborators in the Paris school have argued that a cognitive view of cultural transmission illuminates complex processes underlying the serial transformation and patterned reproduction of ideas and behaviors. While “a pervasive idealization in the study of cultural evolution has been that culture is transmitted only or largely through imitation-based copying,” members of the Paris School have argued that the “mechanisms of cultural propagation are instead many and varied, and often involve re-production, or recurrence, rather than just reproduction” (Claidière et al., 2014, p. 3). On the basis of psychological experiments across a range of experimental subject populations—primarily nonhuman great apes, monkeys, human infants and adults—members of the Paris school argue that biological approaches to cultural evolution overlook the cognitive mechanisms underlying cultural transmission, and as a result, oversimplify processes of cultural evolution for the sake of scientific convenience.

Within the field of cultural evolution, the Paris school has positioned itself as the voice of experimental and psychological approaches to studying cultural transmission. Members of the Paris school agree with biologically-oriented cultural evolutionists on the central aims and terms of debate, but argue that cognitive mechanisms of cultural transmission are more complex than mathematical models of imitation permit. Grounding their research and theories in cognitive universalism also opened another route to antiracism. As Sperber explained,

“There are no relevant biological differences among populations. That doesn’t mean biology is not relevant. What’s relevant to me in biology is what’s common to human beings. How human beings differ is at best a very marginal interest in the kinds of things I am doing. Regarding group differences, there’s good evidence that even the most racist views have very little relevance—they try to get a lot of mileage out of practically nothing. There is every reason not to go there. Scientific reasons, political reasons, there’s an issue of responsibility in research. On the other hand, what human beings have in common, and for instance what they don’t have in common with other animal species—that’s interesting. And that, I think, has a big explanatory role in understanding how humans are capable of culture and why they have the kind of culture they have” (Sperber, personal communication (interview), April 13, 2019).

## Conclusion

In 1950, the United Nations Educational, Scientific and Cultural Organization (UNESCO) published its first Statement on Race. The fight against racism had been enshrined in UNESCO’s constitution and the organization identified the doctrine of racial inequality as one cause of World War II and the Holocaust. In 1949 Alfred Métraux, head of UNESCO’s Race Division, convened a meeting of “experts on race problems” in order to assemble and disseminate scientific facts that would invalidate and dispel race prejudice (Reardon, 2005, p. 26). UNESCO interpreted racism as a problem of doctrine or ideology, “whose solution lay in the liberal principles of scientific education, free communication and exchange, and greater ‘mutual understanding’ between groups of people” (Gil-Riaño, 2023, p. 4). The first Statement on Race was intended to mobilize the authority of science to repudiate Nazi-style scientific racism and ideas of racial hygiene. It asserted the biological unity of the human species and insisted that races could not be ranked as superior or inferior in any hierarchy.

Yet UNESCO’s 1950 Statement on Race elicited a strong backlash from geneticists and physical anthropologists. Although a primary concern of the first Statement’s authors had been delimiting legitimate technical uses of race as a geographic and statistical concept in genetics, prominent geneticists argued that the Statement had gone too far in attempting to undermine all notions of meaningful racial difference. To uphold UNESCO’s commitment to scientific impartiality and rational deliberation, Métraux convened a second committee of physical anthropologists and geneticists, designated this time as “experts on race” (Reardon, 2005, p. 30; Gil-Riaño, 2023, p. 5). In 1951, this second committee published a revised Statement on Race, which agreed with almost all of the claims made in the first Statement: it reaffirmed that all human races have a common evolutionary origin; that racial purity is a myth; that races cannot be classified as superior or inferior; and, that races are not discrete or qualitatively distinct, but should be thought of instead as overlapping populations marked by quantitative and continuous differences of gene frequencies.

But there were two crucial changes to UNESCO’s revised Statement on Race. The first Statement had declared that mental characteristics were not used to scientifically classify races, and that intelligence tests provided no basis for believing that innate differences in intelligence or character existed between races. The second Statement recanted these claims. After the first Statement was published, “’Racial’ intelligence became ground zero in the debate about difference” (Brattain, 2007, p. 1401). Although geneticists and physical anthropologists held a range of views on the topic, many presumed that some racial differences were likely to exist. Sir Ronald Fisher, for example, argued that the Statement should assert that “scientific knowledge provided *a firm basis*, rather than ‘no basis’) for believing that the groups of mankind differ in their innate capacity for intellectual and emotional development” (Brattain, 2007, p. 1404; Reardon, 2005, pp. 30–31). While evidence for this claim did not exist, neither was it required—it reflected a widespread assumption among postwar scientists that placed the burden of proof on those who argued that no racial differences existed. UNESCO’s second Statement on Race reflected this presumption of difference: the revised section on intelligence was deliberately crafted to leave open the possibility that innate and inherited racial differences of intelligence would be discovered.

The second crucial change to UNESCO’s revised Statement on Race was the removal of the claim that biological science supported an ethic of universal human brotherhood. The authors of the second Statement argued that the ethical demand for equal treatment of all human beings did not require scientific evidence that all humans were identical in their genetic and psychological capacities. Some even suggested that the first Statement on Race risked establishing a politically-motivated scientific dogma of equality that reproduced the dynamics of Nazi doctrine albeit with different contents (Reardon, 2005, p. 31; Brattain, 2007, p. 1410; Roberts, 2011, pp. 44–46). Rather than outright reject the possibility of meaningful racial differences then, the authors of the second UNESCO Statement on Race emphasized the conceptual redefinition of race as dynamic, overlapping, and geographically-separated populations differing quantitatively and continuously in gene frequencies. In short, they sought “not to discard race altogether but rather to reform it in the technical terms of population genetics and thus narrow its usage to circumscribed scientific discourses” (Gil-Riaño, 2023, p. 5).

These specific ambivalences of appointed antiracists were exploited precisely by the new scientific racists that emerged in the wake of the War on Poverty and the desegregation of schools in the United States (Brattain, 2007). Shockley and Jensen stepped into the exact openings introduced by the revisions to UNESCO’s second Statement on Race. Jensen fixated on intelligence and culture, and wielded geneticists’ notion of races as populations with ease. He noted that races are “more technically viewed by geneticists as populations having different gene frequencies,” and he even folded postwar population geneticists’ favorite refrain into his scientific racism, writing: “the differences *between* racial groups would account for 23% of the total variance, but—an important point—the differences *within* groups would account for 77% of total variance. When gross socioeconomic level is controlled, the average difference reduces to about 11 IQ points” (1969, pp. 80–81). Jensen also wrote that while research on “the culturally disadvantaged” was replete with environmental explanations for Black children’s performance on IQ tests, the “importance of genetic factors in racial behavioral differences has been greatly ignored, almost to the point of being a tabooed subject” (1969, p. 80). Echoing some of the UNESCO antiracists’ own warnings, he wrote that a “preordained, doctrinaire stance” on human genetic equality presented a “danger to free inquiry” (1969, p. 79).

Even after scientific racists like Shockley and Jensen appropriated postwar antiracists’ vocabularies and virtues to renovate eugenic arguments, liberal scientific institutions avoided direct confrontation. Bodmer and Cavalli-Sforza wrote their article for the *Scientific American* in part because the leaders of the ASHG Social Issues Committee—much like UNESCO’s second committee on race—did not think that they should categorically reject arguments like Jensen’s in an official capacity. They preferred instead to ask individual scientists who were so inclined to write public qualifications (Mitchell, 2017). When Bodmer and Cavalli-Sforza did address Jensen’s eugenic arguments directly, they framed his racism as illogical and as yet unwarranted scientifically. They adopted the stance of scientific skeptics and technical advisors, on site to provide clarification regarding the proper uses of statistics and genetics. They did not outright reject Jensen’s racist assertion of genetically determined racial differences in intelligence, but argued that measured differences in IQ were more likely due to cultural factors. Instead of calling Jensen’s argument what it was—racist, eugenic, and wrong—Cavalli-Sforza laid the foundation for a new field of research to elaborate the claim that culture was more important than race. But like the genetic reconfiguration of race as population, Cavalli-Sforza’s genetic culturalism did not so much refute race as rotate it. If Boasian culturalism handed race off to biology, Cavalli-Sforza’s culturalism tried to offload it again. Yet race remained “the grammar and ghost of population,” as well as culture’s shadow (Murphy, 2017, p. 135; Anderson, 2019, p. 8).

The California and Paris schools of cultural evolution carried Cavalli-Sforza’s postwar antiracism into the twenty-first century, continuing to affirm the evolutionary and cognitive unity of humans in order to sidestep questions of race. They deepened the project of mechanizing culture and quantifying cultural and genetic co-evolution, assuming that a rigorous evolutionary science of culture could effectively obviate scientific racism. Yet as Ghassan Hage (2016, p. 123) writes, despite its long history and some remarkable successes, “it cannot be said that anti-racism has been particularly successful.” In its late twentieth and early twenty-first century forms, cultural evolution exemplifies and illuminates the limits of liberal antiracism.

## Footnotes

1 Cultural Evolution Society. 2016a. “Inaugural Election Results,” p.4: Membership reported as 41.44% based in the US, 13.7% based in the UK, 5.14% based in France, with another 15% from Europe, Canada, and Australia, for a total of more than 75% of members based in these places.

2 These designations of California and Paris Schools of cultural evolution were first introduced by the philosopher of science Kim Sterelny in 2017 to describe the two most prominent approaches to studying cultural evolution in the early twenty-first century.

3 This article is focused on the formation and institutionalization of cultural evolution from 1970 to 2015. It does not attempt to account for developments since 2015 or to capture the breadth of research in the field at present.

4 Tylor did not mention Darwin in the first edition of *Primitive Culture* and mentioned him only once (alongside Herbert Spencer) in the Preface to the second edition, where he wrote: “This absence of particular reference is accounted for by the present work, arranged on its own lines, coming scarcely into contact of detail with the previous works of these eminent philosophers” (Tylor, 1920 [1871], p. vii-viii).

5 R. Sargent Shriver—who was then serving as the head of the Office of Economic Opportunity, leading President Johnson’s War on Poverty, directing the Peace Corps, and funding the construction of the Joseph P. Kennedy Jr. Laboratories in the Department of Genetics at Stanford—repeatedly wrote to the chair of Stanford’s Department of Genetics, Joshua Lederberg, in 1965 to express his concerns about Shockley and to ask Lederberg to respond publicly (Mitchell, 2017, pp. 429–431).
